# Establishing the PM_2.5_-associated inflammatory endotype of chronic rhinosinusitis

**DOI:** 10.1016/j.jaci.2026.02.033

**Published:** 2026-03-04

**Authors:** Daniel H. Lofgren, Christina Dorismond, Rory J. Lubner, Katherine N. Cahill, Justin H. Turner, Rakesh K. Chandra, Mason R. Krysinski, Ping Li, Naweed I. Chowdhury

**Affiliations:** a Department of Otolaryngology–Head and Neck Surgery, Vanderbilt University Medical Center, Nashville; b Department of Allergy, Pulmonary and Critical Care Medicine, Vanderbilt University Medical Center, Nashville; c Department of Otolaryngology–Head and Neck Surgery, University of Alabama Marnix E. Heersink School of Medicine, Birmingham.

**Keywords:** Chronic rhinosinusitis, cytokine, endotype, air pollution

## Abstract

**Background:**

Chronic rhinosinusitis (CRS) is a complex multifactorial disease characterized by persistent sinonasal inflammation of unknown etiology. A unique inflammatory signature of CRS associated with exposure to particulate matter with a diameter of less than 2.5 μm (PM_2.5_) has recently been identified, characterized by univariate elevations in mucus IL-2, IL-5, IL-7, IL-12/23p40, and IL-21.

**Objective:**

We sought to validate and further define this putative endotype by a joint multivariate cytokine analysis.

**Methods:**

Clinical and demographic data for 634 patients undergoing sinus surgery were extracted with a spatiotemporal machine learning model to estimate daily PM_2.5_ exposure for 12 months before surgery. Inflammatory mucus cytokines were quantified with a cytometric bead assay. Levels of IL-2, IL-5, IL-7, IL-13, IL-12/23p40, and IL-21 were log transformed, scaled, and summed to create a composite measure. Spearman correlation and regression analysis were performed to characterize the relationship between this scaled metric and estimated PM_2.5_ exposure.

**Results:**

Estimated 12-month PM_2.5_ levels were positively associated with elevations in the composite cytokine score on univariate analysis (β = 1.17, *P* < .0001). This relationship between the IL-2, IL-5, IL-7, IL-13, IL-12/23p40, and IL-21 composite score and PM_2.5_ levels was persistent after adjusting for numerous potential clinical and sociodemographic confounders, including age, body mass index, history of asthma/allergic rhinitis, polyps, and income/rurality measures (β = 1.27, *P* < .0001).

**Conclusion:**

PM_2.5_-associated CRS is characterized by joint multivariate elevations in mucus IL-2, IL-5, IL-7, IL-12/23p40, IL-13, and IL-21, providing key evidence for a putative mixed type 2 and 3 endotype of CRS associated with air pollution.

Chronic rhinosinusitis (CRS) is a complex multifactorial disease process that significantly reduces patient quality of life and increases health care costs globally.^1–3^ While often classified as CRS with or without nasal polyps, recent emphasis has shifted to instead endotyping CRS by using molecular biomarkers.^2 ,4^ Endotyping has allowed for more targeted and individualized medical therapies for patients, specifically concerning monoclonal antibodies targeting type 2 inflammation, but its full clinical utility is still being investigated.^4–6^ Despite many advances in endotyping, the underlying pathogenesis of CRS remains an area of active investigation.

To this end, there has been a recent increasing focus on the role of environmental exposures on the development of CRS, especially with respect to aerosolized pollutants such as particulate matter with a diameter of less than 2.5 μm (PM_2.5_), which deposit diffusely in the respiratory system.^7–9^ Prior work by our group linked chronic PM_2.5_ exposure in CRS patients with the presence of a mixed type 2–dominant CRS inflammatory response characterized by elevations in IL-2, IL-5, IL-7, IL-12/23p40, IL-13, and IL-21, with elevated PM_2.5_ exposure levels associated with higher tissue eosinophil counts and higher odds of comorbid asthma.^8^ Other studies have also examined PM_2.5_ exposure in CRS and have identified links with airway inflammation, poorly controlled asthma, recalcitrant CRS requiring surgery, and reduced 22-Item Sinonasal Outcomes Test (SNOT-22) total score change after endoscopic sinus surgery (ESS).^7,9,10^

A previous study by our group evaluated the ability of mucus cytokines to define a putative type 2–high CRS endotype defined by IL-5 and IL-13 scaled scores in control patients using a 2–standard deviation (SD) cutoff.^11^ Compared to those with low type 2 disease, patients with high type 2 CRS had higher tissue eosinophilia, more prior sinus procedures, worse preoperative computed tomography scores, and more severe clinical disease. Our initial research into PM_2.5_-associated inflammatory cytokines in CRS only looked at single cytokines’ relationships and had a small sample size, so we aimed to adapt our approach to explore and define the concept of a PM_2.5_-linked inflammatory endotype characterized by joint elevations across IL-2, IL-5, IL-7, IL-12/23p40, IL-13, and IL-21, as well as assess relationships between this composite measure and clinical characteristics. A secondary goal of our study was to replicate our initial findings in a larger cohort of patients to provide additional validation of our hypothesis. By utilizing a composite score approach similar to our prior work in type 2 inflammation, we sought to better understand the nature of this set of inflammatory cytokines, how they may be influenced by both PM_2.5_ and patient factors, and how best to characterize the clinical impact of this putative endotype of disease.

## METHODS

### Enrollment and inclusion/exclusion criteria

This single-institution prospective cohort study was reviewed and approved by the institutional review board of Vanderbilt University Medical Center. Data were stored using an encrypted online Health Insurance Portability and Accountability Act–compliant database (REDCap, Vanderbilt University, Nashville, Tenn). Patients who sought care at the Vanderbilt University Medical Center Rhinology clinic and the Vanderbilt Asthma, Sinus, and Allergy Program with bilateral CRS who underwent ESS between 2015 and 2024 were included. Exclusion criteria included patients with systemic steroid receipt within 4 weeks of surgery, cystic fibrosis, or known autoimmune disease. A diagnosis of CRS was made using the International Consensus Statement on Allergy and Rhinology: Rhinosinusitis and the American Academy of Otolaryngology–Head and Neck Surgery Clinical Practice Guidelines.^2^ Allergic rhinitis was determined by the presence of cardinal symptoms including congestion, nasal drainage, sneezing, or itching in conjunction with positivity on skin prick or radioallergosorbent testing. All patients underwent ESS after disease failed to respond during an adequate trial of medical management, including topical nasal corticosteroids, nasal saline irrigations, and oral steroids and antibiotics. Control patients were recruited from the population of adult patients undergoing endoscopic skull base surgery for nonsecretory pituitary adenomas or cerebrospinal fluid rhinorrhea. Radiographic disease severity was quantified for CRS patients using the Lund-Mackay score, and patients were also administered the SNOT-22 before surgery. Tissue eosinophil counts were recorded from histopathologic slides as an average over 5 high-power fields by a board-certified head and neck pathologist.

### Middle meatal cytokine quantification and scaling

Before initiating ESS, two 9 × 24 mm polyurethane sponges (Summit Medical, St Paul, Minn) were placed into the middle meatus of each subject under direct endoscopic visualization and left for 5 minutes. Cytokines were then quantified as previously described.^8,11^ Briefly, sponges were centrifuged at 14,000 × *g* for 10 minutes to elute mucus and vortexed, with supernatants separated and frozen at −80°C for pooled analysis. Cytokine assays were then performed using a standard sensitivity multiplex cytokine bead assay (BD Biosciences, Franklin Lakes, NJ). Data were analyzed by BD FCAP Array v3.0 software. Levels of 21 cytokines/chemokines (IL-1α, IL-1β, IL-2, IL-3, IL-4, IL-5, IL-6, IL-7, IL-8, IL-9, IL-10, IL-12/23p40, IL-13, IL-17A, IL-18, IL-21, TNF-α, IFN-γ, eotaxin, RANTES, GM-CSF) were quantified, and raw counts were log normalized.

### Geospatial estimation of preoperative 1-year PM_2.5_ levels

Patient home addresses were converted to latitude and longitude coordinates using ‘geocoder’ in the DeGAUSS tool kit.^12^ Coordinates were then used to obtain tract-level income and rurality measures. A validated spatiotemporal machine learning model with 0.75 km^2^ resolution was then used to obtain estimated daily PM_2.5_ levels for the entire year before each patient’s surgery date and averaged as previously published.^13^

### Statistical methods

Baseline differences between CRS and control patients were assessed by the Wilcoxon rank sum test for continuous variables and the Pearson chi-square test for categorical variables. Normalized cytokine levels were then regressed against 12-month PM_2.5_ levels to characterize the relationship between mucus cytokines and PM_2.5_, accounting for potential confounders of age, body mass index (BMI), asthma, allergic rhinitis, polyps, income, and rurality. We then took cytokine levels of IL-2, IL-5, IL-7, IL-12/23p40, IL-13, and IL-21 and standardized these values by subtracting the mean and dividing by the SD of each cytokine to create a unitless scaled score. Each cytokine scaled score was then summed to create a composite multicytokine scaled inflammation score. A similar approach was then used for cytokine data from control patients, with a cutoff threshold for the composite score defined at 2 SDs above the mean in controls. We then used the control-derived composite cytokine cutoff to classify disease into high and low mixed type 2 groups using a previously published approach by our group.^11^ Logistic and linear regression models were then used to examine the association of both the high/low mixed type 2 endotype and the continuous cytokine score with PM_2.5_ levels after adjustment for confounders. Finally, we examined the relationship of the composite cytokine score with various clinical features, including radiographic disease severity, tissue eosinophil counts, asthma, nasal polyposis, and prior surgery. A prespecified alpha threshold of .05 was used to denote a statistically significant result on all hypothesis tests.

### Results

Clinical and demographic characteristics of our patient group are presented in Table I. Total patient enrollment included 634 participants with CRS and 88 control patients, with 336 (53%) of CRS patients presenting with nasal polyps. There was a high prevalence of comorbid allergic rhinitis (57.5%) and asthma (43.8%) in the CRS patient cohort, with a 1-year mean PM_2.5_ level of 8.1 6 0.6 μg/m^3^. Control patients were notably different across baseline measures of age, sex, BMI, allergic rhinitis, and asthma, with a slightly lower but statistically significant difference in average preoperative PM_2.5_ levels (8.0 ± 0.5 μg/m^3^, *P* = .02; Table I).

### Single mucus cytokine regression analysis

We initially utilized our previously published approach to model each cytokine level in the panel as a function of mean 12-month preoperative PM_2.5_ levels. This was adjusted for the potential confounders of age, BMI, asthma, allergic rhinitis, polyps, income, and rurality, with the intent of validating prior findings in this larger cohort. Statistically significant coefficients from each model are presented in Table II. Our overall findings largely mirrored the results from our initial cohort, with statistically positive relationships between IL-2, IL-7, IL-12/23p40, IL-13, and IL-21. Only IL-5 was noted to be nonsignificant in the larger group (*P* = .17).

### Joint cytokine scaled score regression analysis

**FIG 1.**
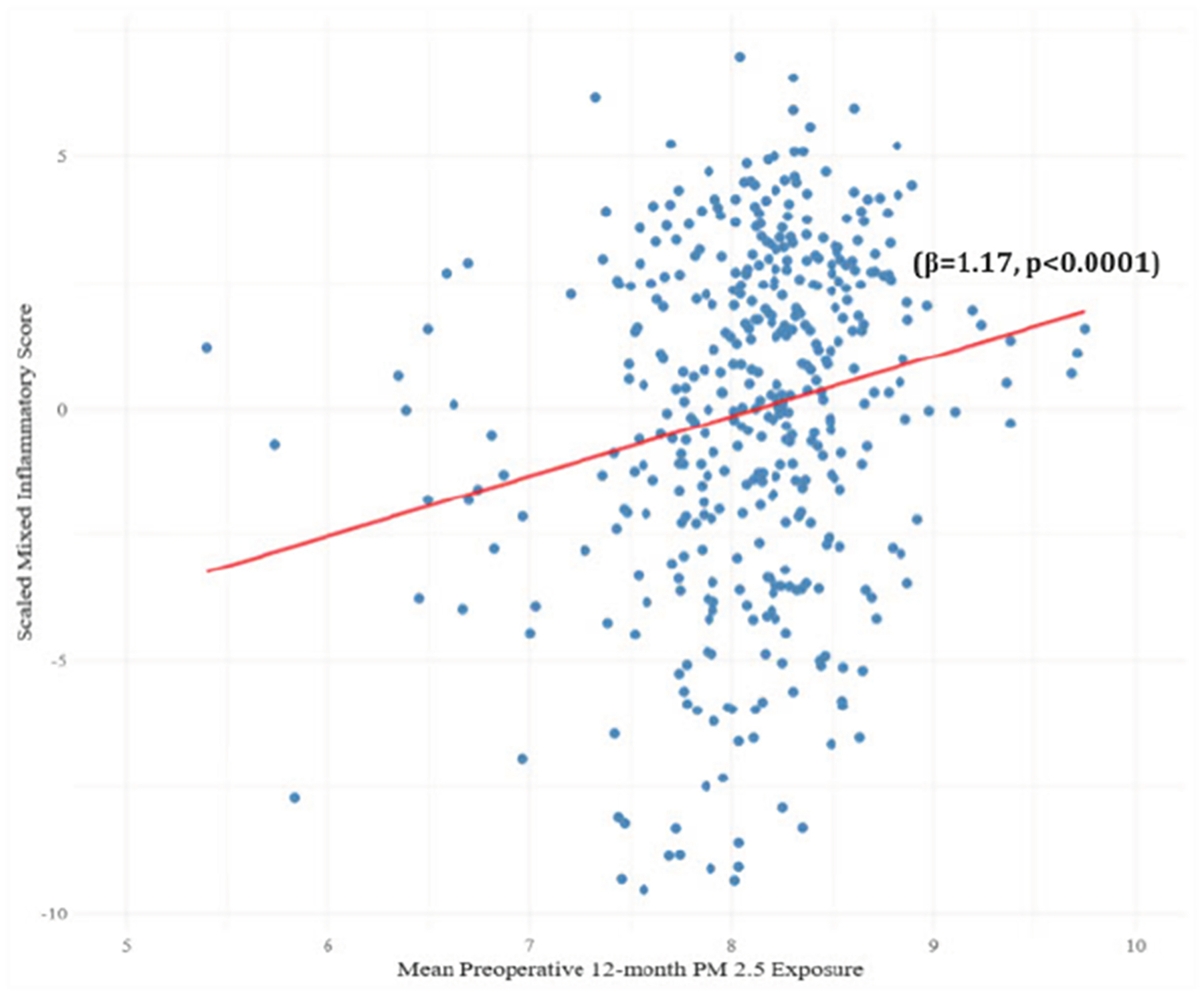
Scatterplot of scaled composite mixed type 2 inflammatory score and PM_2.5_ levels in CRS patients, with univariate regression line estimating the relationship between the combined cytokine score as a function of mean 12-month PM_2.5_ estimates. β indicates log change in scaled mixed cytokine score per 1 μg/m^3^ change in 12-month PM_2.5._

We next sought to create a composite measure of inflammation characterized by joint elevations in IL-2, IL-5, IL-7, IL-12/23p40, IL-13, and IL-21 on the basis of our prior work. Of note, IL-5 was kept in the joint score despite being nonsignificant in our single cytokine model on the basis of its known biologic role in the type 2 inflammatory cascade alongside IL-13. Raw cytokine levels were log normalized, scaled, and centered to create standardized cytokine scores that were then summed to form a composite score. There was a statistically significant positive bivariate correlation between estimated exposures and elevations in the composite cytokine score (Rs = 0.20, *P* < .0001). To further quantify this relationship, a univariate unadjusted regression model was fit between preoperative PM_2.5_ levels and the composite scaled cytokine score, again showing a statistically significant positive relationship (β = 1.17, *P* < .0001, Fig 1). Finally, we adjusted this univariate model with a multivariate regression model accounting for age, BMI, asthma, allergic rhinitis, polyps, income, and rurality, and noted that 12-month PM_2.5_ exposures remained statistically significant with a comparable magnitude (β = 1.27, *P* < .0001). Notably, the presence of nasal polyps was also significantly associated with this score (β = 1.84, *P* < .0001), but a history of asthma (β = 20.08, *P* = .79) and allergic rhinitis (β = 0.10, *P* = .76) were not linked. Results from the full model are seen in Table III.

### Comparison relative to control patient data

Next we used mucus cytokine levels from our control population to determine a threshold value for a high level of inflammation in the composite cytokine score. A similar process was used to log-normalize, standardize, and sum levels of IL-2, IL-5, IL-7, IL-12/23p40, IL-13, and IL-21 from control patients, with a cutoff set at 2 SDs (95th percentile) beyond the mean value for the composite inflammatory cytokine score. We then stratified CRS patients according to the cutoff score into high and low mixed type 2 inflammation groups. We used multivariate logistic regression to estimate the odds of having high mixed inflammation after adjusting for age, BMI, asthma, allergic rhinitis, polyps, income, and rurality. The model estimated a higher likelihood of being in the high mixed inflammatory group with increasing PM_2.5_ exposure levels (β = 0.441; odds ratio [OR] 1.55, *P* = .031). There were notably no other statistically significant predictors of the high mixed type 2 endotype.

### Association with baseline and perioperative clinical features in CRS

As a final step in the analysis, we looked at associations between several clinical features of CRS patients and the composite mixed type 2 score. On logistic regression, higher mixed type 2 score levels were associated with higher odds of prior surgery (OR = 1.08, *P* = .018), asthma (OR = 1.06, *P* =.04), and nasal polyposis (OR = 1.19, *P* < .0001), but not allergic rhinitis (OR = 1.03, *P* = .19). Mixed type 2 composite scores were also significantly associated with tissue eosinophil counts (β 5 1.66, *P* = .015), but they were not linked to baseline Lund-Mackay scores (β = 0.148, *P* = .23) or total preoperative SNOT-22 scores (β = 20.046, *P* = .89).

## DISCUSSION

This study investigated the relationship between PM_2.5_ exposure and inflammatory markers in patients with CRS compared to controls in order to characterize a PM_2.5_-associated inflammatory endotype using an approach our group previously used to investigate type 2 cytokines in CRS.^11^ We also sought to validate our prior work demonstrating an association of IL-2, IL-5, IL-7, IL-12/23p40, and IL-21 with 12-month PM_2.5_ exposure on a multivariate single cytokine regression analysis.^8^ Notably, these associations remained generally consistent in the larger sample, with statistically positive relationships between IL-2, IL-7, IL-12/23p40, IL-13, and IL-21; only IL-5 from our previous study was nonsignificant, replaced by IL-13 in the larger sample.

Because these 6 cytokines are not exclusively within one of the 3 canonical inflammatory patterns identified in mammals, we hypothesize that the relatively consistent relationships seen across both studies may be sign of activation across multiple pathways, with the type 2 program being the predominant feature given the inclusion of two of its prototypical cytokines (IL-5 and IL-13) and the association of eosinophil counts with this inflammatory signature. These results are similar to findings seen in a mouse model of CRS with short-term particulate matter exposure, which identified elevations in several type 2 and 3 cytokines in *Nrf2*^−*/*−^ mice experimentally exposed to 14 days of PM_2.5_; however, we did not see elevations of IL-17A in our patient population with estimated exposures.^14^ While not considered a classic type 3 cytokine, the IL-12/23p40 subunit is known to be involved in type 3 inflammatory responses in other disease states and may also be a biomarker of type 3 activity, as it relates to chronic air pollution exposure in CRS.^15^ Additionally, there is evidence for the role of IL-21 as a stimulator of both T_H_17 cells and type 3 innate immune cells, thus linking IL-21 to the type 3 inflammatory program.^16 ,17^ Importantly, a recent study in a mouse model of eosinophilic CRS implicated IL-21 as a key cytokine inducing both type 2 and 3 inflammation, with both T_H_2 and T_H_17 cells identified as the source of IL-21 in human nasal samples.^18^ IL-7 may also have a role in promoting type 2 inflammation through memory T_H_2 cells in CRS with nasal polyps, leading to persistent inflammatory memory and potentially treatment recalcitrant disease.^19^ In contrast to the other cytokines, IL-2 has less evidence for a specific role in CRS. IL-2 is a noted general T-cell growth factor that is thought to play a functional role in the maintenance of regulatory T cells as well as promoting the proliferation and expansion of effector cells and innate immune cells. Taken together, the elevations in these cytokines with PM_2.5_ exposure appear to be most consistent with a mixed type 2 and 3 inflammatory process.

The notion of a unique PM_2.5_-associated inflammatory signature is further supported by our composite score model, which was independently associated with estimated PM_2.5_ exposure even after accounting for age, BMI, asthma, allergic rhinitis, polyps, income, and rurality (Table III). This relationship was also seen when using control cytokine data to define a high mixed inflammatory cutoff for CRS patients and modeling exposures as a function of this, with an adjusted OR of 1.55 per 1 μg/m^3^ of 12-month PM_2.5_. Stated differently, each 1 μg/m^3^ increase in preoperative annual PM_2.5_ exposure increases the probability of being in the high mixed inflammatory group by 60.7%, and this relationship was unique to PM_2.5_ in the model, as other covariates were not statistically significant.

Finally, we investigated potential links between the composite inflammation score and various measures of clinicopathologic and radiographic markers of disease severity. There were noted statistically significant relationships between the composite PM_2.5_ score and tissue eosinophil counts, previous sinus surgery, asthma, and nasal polyps, suggesting that these factors could potentially be influenced by chronic air pollution exposure. Eosinophilic infiltration has been noted in several studies of PM_2.5_ exposure in mice, lending credence to our human findings, and the link between air pollution and asthma is well established.^14,15^ Interestingly, there was a small but statistically notable association between the composite PM_2.5_ score and having revision surgery, indicating that there may be an impact on clinical outcomes. A recent study showed that higher perioperative PM_2.5_ exposure was associated with reduced SNOT-22 improvement after surgery, and the frequency of outpatient visits for CRS also appears to be increased in conjunction with higher exposures.^9,20^ Presuming that there is a notable type 3 component to the inflammatory milieu of CRS in the setting of air pollution, this finding is certainly plausible, given the evidence for reduced medical and surgical treatment responsiveness in mixed type 2 and 3 CRS endotypes.^21,22^ Taken together, the above findings are the first in humans to exclusively link PM_2.5_ with this unique mixed inflammatory axis; our findings also demonstrate potential clinical implications for this endotype of disease that warrant research into targeted treatments.

Several limitations should be noted for our study. First, the recruitment of patients from a single academic medical center in the southeastern United States with a predominantly White demographic may limit the generalizability of the results. Future research should involve multiregional or multicenter collaborations to increase population size and diversity, addressing geographic limitations. Second, the study population may be biased, as we only recruited CRS patients with disease that was refractory to medical therapy and who had undergone surgery, excluding those with medically responsive CRS. Third, our approach does not account for environmental exposures beyond PM_2.5_. Given that other pollutants like ozone, carbon monoxide, lead, sulfur dioxide, and nitrogen dioxide are known to contribute to human morbidity and mortality, future models incorporating data on these additional pollutants could provide a more comprehensive understanding of this subject.^8 ,10,23–25^ Furthermore, the model relies on estimated outdoor exposures that are based on home addresses, which may not fully capture a patient’s total exposure, neglecting factors like occupation, travel, or indoor air pollution sources.^26^ Finally, our cytometric bead assay panel was limited to the selected cytokines in the multiplex kit, which may not capture all chemokine subtypes found in nasal mucus, such as CCL24 and CCL26.^27^ Future research could explore the use of wearable devices to track pollutant exposure at a more personalized level, encompassing the entirety of each patient’s exposome.^28^

In conclusion, PM_2.5_-associated inflammation in CRS appears to be characterized by joint global elevations in mucus IL-2, IL-5, IL-7, IL-12/23p40, IL-13, and IL-21. Further experimental validation and investigation of upstream pathways are needed to identify possible mechanisms leading to this endotype as well as to discover potential targeted therapeutics.

## Figures and Tables

**TABLE I.** Baseline characteristics of patients

Characteristic	CRS (n = 634)	Control (n = 88)	*P* value	Overall (N = 722)
Age (years)	49.3 (15.9) [18.0–82.0]	53.8 (15.1) [18.0–78.0]	.014*	49.8 (15.8) [18.0–82.0]
Sex			.015*	
Female	288.0 (45.6%)	49.0 (59.8%)		337.0 (47.2%)
Male	344.0 (54.4%)	33.0 (40.2%)		377.0 (52.8%)
Race			.031*	
White	537.0 (85.2%)	60.0 (72.3%)		597.0 (83.7%)
African American	65.0 (10.3%)	19.0 (22.9%)		84.0 (11.8%)
Hispanic	10.0 (1.6%)	2.0 (2.4%)		12.0 (1.7%)
Asian	8.0 (1.3%)	1.0 (1.2%)		9.0 (1.3%)
Other	6.0 (1.0%)	1.0 (1.2%)		7.0 (1.0%)
Unknown	4.0 (0.6%)	0		4.0 (0.6%)
Body mass index (kg/m^2^)	29.5 (7.0) [16.7–77.3]	34.7 (7.6) [22.1–50.2]	<.001*	30.1 (7.3) [16.7–77.3]
Asthma			<.001*	
No	355.0 (56.2%)	75.0 (90.4%)		430.0 (60.1%)
Yes	277.0 (43.8%)	8.0 (9.6%)		285.0 (39.9%)
Allergic rhinitis			<.001*	
No	269.0 (42.5%)	71.0 (85.5%)		340.0 (47.5%)
Yes	364.0 (57.5%)	12.0 (14.5%)		376.0 (52.5%)
12-month mean PM_2.5_ (μg/m^3^)	8.1 (0.6) [5.4–13.0]	8.0 (0.5) [6.6–9.1]	.020*	8.1 (0.6) [5.4–13.0]

Data are presented as means (SDs) [ranges] or as nos. (%).

* Statistically significant (*P* < .05) by Wilcoxon rank sum test, Pearson chi-square test, or Fisher exact test.

**TABLE II.** Single cytokine multivariate models

Cytokine	β(PM_2.5_)	SE	*t* test statistic	*P* value
IL-1α†	0.067	0.221	0.307	.75
IL-1β‡	−0.132	0.188	−0.705	.48
IL-2	0.831	0.196	4.237	<.00001*
IL-4§	0.187	0.162	1.15	.25
IL-5§	0.194	0.142	1.36	.17
IL-6	−0.034	0.086	−0.406	.685
IL-7§	0.286	0.142	2.003	.045*
IL-8	−0.055	0.082	−0.678	.49
IL-9§	0.277	0.214	1.293	.19
IL-10§	0.052	0.142	0.366	.714
IL-12/23p40	0.343	0.144	2.042	.041*
IL-13§	0.453	0.199	2.273	.023*
IL-17A¶	−0.05	0.173	−0.292	.77
IL-21	0.403	0.187	2.152	.032*
TNF-α	0.081	0.191	0.424	.672
IFN-γ	−0.288	0.161	−1.792	.073
Eotaxin‡	0.011	0.126	0.085	.932
RANTES†	0.086	0.168	0.514	.608
GM-CSF	0.386	0.251	1.538	.125

β indicates effect estimate per 1 μg/m^3^ change in 12-month PM_2.5_

*GM-CSF,* Granulocyte-macrophage colony-stimulating factor; *RUCA,* Rural Urban Commuting Area; *SE,* standard error.

† Significant covariates include age and polyp status.

‡ Significant covariates include age.

§ Significant covariates include polyp status.

¶ Significant covariates include polyp status and asthma.

* Statistically significant (*P* < .05). Multivariate models adjusted for age, body mass index, income, RUCA score, nasal polyps, allergic rhinitis, and asthma.

**TABLE III.** Composite inflammatory score multivariate model

Variable	β	SE	*t* test statistic	*P* value
PM_2.5_	1.26	0.293	4.32	<.00001*
AGI	−0.003	0.003	−1.039	.29
RUCA	0.030	0.085	0.354	.724
Age	−0.018	0.011	−1.674	.09
BMI	−0.003	0.024	−0.124	.90
Asthma	−0.089	0.346	−0.258	.796
Allergic rhinitis	0.106	0.345	0.308	.758
Nasal polyps	1.84	0.349	5.268	<.00001*

β indicates effect estimate per 1 unit change for continuous variables and for presence of categorical variables.

*AGI,* Adjusted gross income; *RUCA,* rural–urban commuting area; *SE,* standard error.

* Statistically significant (*P* < .05).

## Key messages

- We characterized the inflammatory profile of PM_2.5_-associated CRS in a large CRS patient population and compared to healthy control patients.
- This T H 2-predominant mixed endotype is characterized by joint global elevations in IL-2, IL-5, IL-7, IL-12/23p40, IL-13, and IL-21.
- This endotype was only significantly associated with PM_2.5_ exposure, and these patients had statistically significantly higher rates of asthma, nasal polyps, tissue eosinophils, and prior surgery.

## Abbreviations used

BMI: Body mass index
CRS: Chronic rhinosinusitis
ESS: Endoscopic sinus surgery
OR: Odds ratio
PM_2.5_: Particulate matter less than 2.5 μm in diameter
RANTES: Regulated on activation, normal T-cell–expressed and secreted
SD: Standard deviation
SNOT-22: 22-Item Sinonasal Outcomes Test
